# Third-generation sequencing and metabolome analysis reveal candidate genes and metabolites with altered levels in albino jackfruit seedlings

**DOI:** 10.1186/s12864-021-07873-y

**Published:** 2021-07-16

**Authors:** Xiangxu Meng, Jiahong Xu, Maoning Zhang, Ruyue Du, Wenxiu Zhao, Qing Zeng, Zhihua Tu, Jinhui Chen, Beibei Chen

**Affiliations:** 1 Key Laboratory of Genetics and Germplasm Innovation of Tropical Special Forest Trees and Ornamental Plants, Ministry of Education/Engineering Research Center of Rare and Precious Tree Species in Hainan Province, School of Forestry, Hainan University, 570228 Haikou, People’s Republic of China; 2 Hainan Key Laboratory for Biology of Tropical Ornamental Plant Germplasm, Institute of Tropical Agriculture and Forestry, School of Forestry, Hainan University, 570228 Haikou, People’s Republic of China; 3 School of Agricultural Sciences, Zhengzhou University, 450001 Zhengzhou, People’s Republic of China; 4 College of Coastal Agricultural Sciences, Guangdong Ocean University, 524088 Zhanjiang, People’s Republic of China

**Keywords:** Albino mutants, *Artocarpus heterophyllus*, Carbon fixation, Differentially expressed genes, High-throughput sequencing, Significantly changed metabolites, Third-generation sequencing

## Abstract

**Background:**

Most plants rely on photosynthesis; therefore, albinism in plants with leaves that are white instead of green causes slow growth, dwarfing, and even death. Although albinism has been characterized in annual model plants, little is known about albino trees. Jackfruit (*Artocarpus heterophyllus*) is an important tropical fruit tree species. To gain insight into the mechanisms underlying the differential growth and development between albino jackfruit mutants and green seedlings, we analyzed root, stem, and leaf tissues by combining PacBio single-molecule real-time (SMRT) sequencing, high-throughput RNA-sequencing (RNA-seq), and metabolomic analysis.

**Results:**

We identified 8,202 differentially expressed genes (DEGs), including 225 genes encoding transcription factors (TFs), from 82,572 full-length transcripts. We also identified 298 significantly changed metabolites (SCMs) in albino *A. heterophyllus* seedlings from a set of 692 metabolites in *A. heterophyllus* seedlings. Pathway analysis revealed that these DEGs were highly enriched in metabolic pathways such as ‘photosynthesis’, ‘carbon fixation in photosynthetic organisms’, ‘glycolysis/gluconeogenesis’, and ‘TCA cycle’. Analysis of the metabolites revealed 76 SCMs associated with metabolic pathways in the albino mutants, including L-aspartic acid, citric acid, succinic acid, and fumaric acid. We selected 225 differentially expressed TF genes, 333 differentially expressed metabolic pathway genes, and 76 SCMs to construct two correlation networks. Analysis of the TF–DEG network suggested that basic helix-loop-helix (bHLH) and MYB-related TFs regulate the expression of genes involved in carbon fixation and energy metabolism to affect light responses or photomorphogenesis and normal growth. Further analysis of the DEG–SCM correlation network and the photosynthetic carbon fixation pathway suggested that *NAD-ME2* (encoding a malic enzyme) and L-aspartic acid jointly inhibit carbon fixation in the albino mutants, resulting in reduced photosynthetic efficiency and inhibited plant growth.

**Conclusions:**

Our preliminarily screening identified candidate genes and metabolites specifically affected in albino *A. heterophyllus* seedlings, laying the foundation for further study of the regulatory mechanism of carbon fixation during photosynthesis and energy metabolism. In addition, our findings elucidate the way genes and metabolites respond in albino trees.

**Supplementary Information:**

The online version contains supplementary material available at 10.1186/s12864-021-07873-y.

## Background 

Albino mutations are common in the plant kingdom. In albino tea mutants, the significantly changed genes and metabolites were enriched in photosynthesis and starch and sucrose metabolic pathways after plant albinism [1]. In albino leaf tissue of *Hydrangea macrophylla*, combined metabolome and transcriptome analyses revealed the changed genes and metabolites were significantly enriched in the chlorophyll synthesis pathway and TCA cycle in response to albinism [2]. In *Arabidopsis thaliana* albino mutants, the altered genes and metabolites after plant albinism were mainly involved in the TCA cycle and the oxidative pentose phosphate pathway in response to albinism [3]. In addition, Tang et al. confirmed that the *OsPPR6* gene is responsible for the albino mutant phenotype of rice through transgenic experiments [4]. Yu et al. obtained albino lethal mutants of *A. thaliana* at the seedling stage by knocking out the *AtECB2* gene [5]. These findings indicate that albinism affects photosynthesis and energy metabolism, thereby hindering plant growth and development. However, studies of albinism in tropical woody plants are lacking.

*Artocarpus heterophyllus* (jackfruit) is an important tropical fruit tree species that is widely planted in various countries such as Brazil, Thailand, Indonesia, Malaysia, and China [6]. Jackfruit is grown for its sweet-tasting fruit and for its wood [7]. We accidentally discovered albino mutants under an *A. heterophyllus* tree. These mutants are unable to grow normally and die prematurely and therefore do not produce fruit and wood. To date, only a few reports are available about the morphological and physiological characteristics of *A. heterophyllus* albino mutants [8, 9]. These albino mutants represent an excellent material for studying photosynthesis and metabolic processes in woody plants.

Multi-omics technologies are effective methods for investigating the responses of plants under stress. Combined transcriptome and metabolome analysis and co-expression network analysis have been widely used to reveal the molecular mechanisms underlying biochemical processes and to identify key genes and metabolites [10–12]. Single-molecule real-time (SMRT) sequencing combined with Illumina sequencing is used to generate high-quality full-length transcripts, reduce the mis-assembly of genes, and enhance the accuracy of transcriptome data [13–15]. Metabolomics, like transcriptomics, is an important tool for systematic biology, providing insight into the ongoing intracellular activities regulated by metabolites, such as energy transfer and cell signaling [16, 17]. Therefore, the integration of metabolomics and transcriptomics can provide a system-wide understanding of the transcriptomic and metabolic changes in *A. heterophyllus* seedlings in response to albinism.

In this study, we performed combined metabolome and transcriptome analysis in root, stem, and leaf tissues of *A. heterophyllus* albino mutants and green seedlings, providing a broad overview of their metabolic and transcriptional differences. The results of this study enrich plant databases, improve our understanding of candidate genes and metabolites after plant albinism, and provide a foundation for the study of tropical fruit trees.

## Results

### Analysis of transcriptome data

To reveal the changes in gene expression in albino *A. heterophyllus* seedlings compared with green seedlings, we sequenced RNA pools from these seedlings and analyzed them using the PacBio Sequel platform. Numerous accurate long reads were obtained. A total of 411,622 polymerase reads (average read length of 46,829 bp) and 8,047,651 subreads (average read length of 2,320 bp) were produced with SMRT (Table 1). To provide more accurate sequence information, 347,472 circular consensus sequences (CCSs; average read length of 2,890 bp) were obtained from subreads that required at least two full-pass subreads in each insertion sequence (Table 1). SMRTlink identified 305,585 full-length reads and 304,319 full-length non-chimeric (FLNC) reads (average read length of 2,684 bp) (Table 1). The FLNC reads of the same transcript were clustered, and redundant reads were removed to obtain consensus reads using the ICE algorithm. Non-full-length non-chimeric reads were used to correct the consensus reads using arrow software, and 153,209 polished consensus sequences were ultimately obtained, with a mean length of 2,723 bp (Table 1).

**Table 1:** Summary of reads from third-generation sequencing

Item	Number	Mean length
**Polymerase reads**	**411,622**	**46,829**
**Subreads**	**8,047,651**	**2,320**
**Circular consensus sequences (CCS)**	**347,472**	**2,890**
**Full-length non-chimeric reads (FLNC)**	**304,319**	**2,684**
**Polished consensus reads**	**153,209**	**2,723**
**Corrected polished consensus reads**	**153,209**	**2,720**
**Full-length transcripts (genes)**	**82,572**	**2,889**

The 18 cDNA libraries were sequenced following the Illumina HiSeq 2500 platform paired-end protocol. RNA-seq generated 172,744,942 (AhCr), 166,037,836 (AhWr), 189,054,956 (AhCs), 154,369,546 (AhWs), 177,232,708 (AhCf), and 167,482,428 (AhWf) raw reads. After trimming, 166,805,876 (AhCr), 161,897,400 (AhWr), 183,062,028 (AhCs), 151,763,044 (AhWs), 173,309,570 (AhCf), and 160,915,478 (AhWf) clean reads remained (Table 2).

**Table 2:** Summary of RNA-seq data for all samples

Samples		Raw reads	Clean reads	Clean bases (G)
**AhCr**	**Average**	**57,581,647.33**	**55,601,958.67**	**8.34**
	**Total**	**172,744,942**	**166,805,876**	**25.02**
**AhWr**	**Average**	**55,345,945.33**	**53,965,800**	**8.09**
	**Total**	**166,037,836**	**161,897,400**	**24.28**
**AhCs**	**Average**	**63,018,318.67**	**61,020,676**	**9.15**
	**Total**	**189,054,956**	**183,062,028**	**27.46**
**AhWs**	**Average**	**51,456,515.33**	**50,587,681.33**	**7.59**
	**Total**	**154,369,546**	**151,763,044**	**22.77**
**AhCf**	**Average**	**59,077,569.33**	**57,769,856.67**	**8.67**
	**Total**	**177,232,708**	**173,309,570**	**26**
**AhWf**	**Average**	**55,827,476**	**53,638,492.67**	**8.05**
	**Total**	**167,482,428**	**160,915,478**	**24.14**

We used the clean reads to correct the polished consensus sequences produced by third-generation sequencing with the PacBio Sequel platform. A total of 82,572 full-length transcripts were obtained with SMRT technology and used as reference (ref) sequences for the genes (Table 1). The clean reads of each sample were mapped to the ref sequences. The number of mapped reads in the 18 libraries ranged from 38,483,936 to 39,026,306, and the mapping ratios ranged from 77.55 to 85.16 % (Supplementary Table S1).

### Identification and cluster analysis of DEGs

**Fig. 1:**
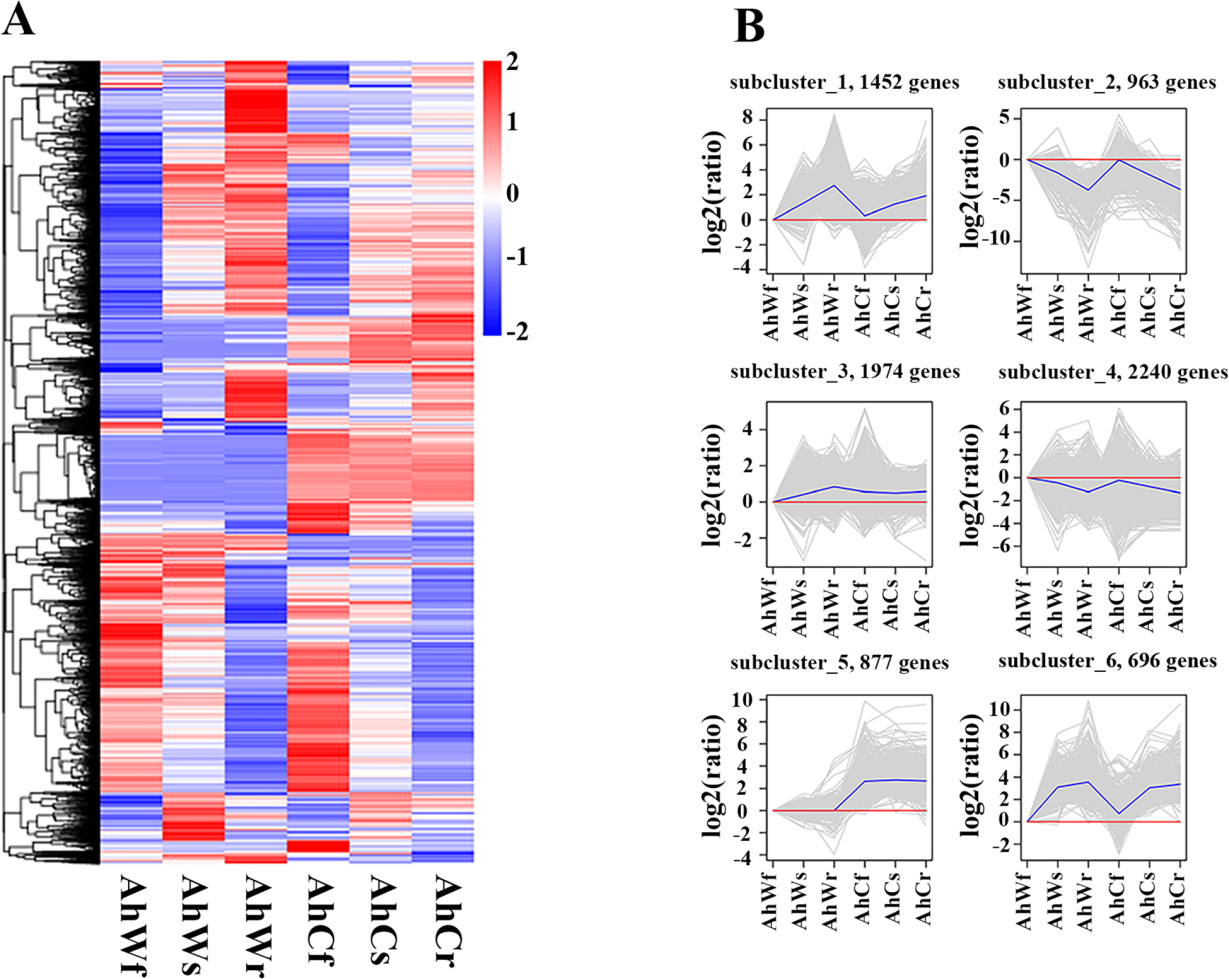
Clustering analysis of DEGs between albino and green *A. heterophyllus* seedlings. **A** Heat map of 8,202 DEGs based on hierarchical clustering analysis. Upregulated (red) and downregulated (blue) genes are shown. **B** The 8,202 DEGs were grouped into six subclusters. The number of genes per cluster is shown at the top of each cluster. Blue lines represent the average relative expression levels of DEGs in each subcluster; gray lines represent the relative expression levels of each gene in each cluster

To investigate the global differences in the transcriptome dynamics between the albino mutants and green seedlings, we identified 1,903 (AhWr vs. AhCr), 1,134 (AhWs vs. AhCs), 894 (AhWf vs. AhCf), 92 (AhCs vs. AhCf), 77 (AhCr vs. AhCs), 1,139 (AhCr vs. AhCf), 1,465 (AhWs vs. AhWf), 2,419 (AhWr vs. AhWs) and 5,552 (AhWr vs. AhWf) DEGs based on the criteria |log_2_FC| ≥ 1 and *q*-value < 0.05 (Supplementary Table S2). After removing the repetitive genes, total of 8,202 DEGs were obtained (Fig. 1 A). The 8,202 DEGs were grouped into six subclusters with various expression patterns using the hierarchical clustering algorithm (Fig. 1B). Genes in cluster 1 (1,452 genes), cluster 3 (1,974 genes), and cluster 6 (696 genes) were upregulated in all samples (Fig. 1B). Genes in cluster 1 and cluster 6 had similar expression patterns; they were strongly expressed in AhWf, AhWs, and AhWr and expressed at low levels in AhCf, AhCs, and AhCr (Fig. 1B). Genes in cluster 3 were all upregulated in both albino mutant and green seedlings (Fig. 1B). GO enrichment analysis revealed that most genes in cluster 3 and cluster 6 were associated with oxidation-reduction processes and oxidoreductase activity (Supplementary Fig. S1C, F). KEGG enrichment analysis indicated that genes in cluster 1 were associated with the glycolysis/gluconeogenesis pathway (Supplementary Fig. S2A).

Genes in cluster 2 (963 genes) and cluster 4 (2,240 genes) were expressed at low levels in AhCf, AhCs, and AhCr (Fig. 1B). Most DEGs in cluster 2 and cluster 4 were associated with photosynthesis and carbon fixation in photosynthetic organisms (Supplementary Fig. S2B, D). However, the expression patterns of the 877 genes in cluster 5 were unusual: they were not expressed in AhWf, AhWs, or AhWr but were strongly expressed in AhCf, AhCs, and AhCr (Fig. 1B). Functional analysis of these genes showed that they were related to the negative regulation of peptidase activity and negative regulation of programmed cell death (Supplementary Fig. S1E).

### Metabolic differences between the leaves of albino mutants and green seedlings

**Fig. 2:**
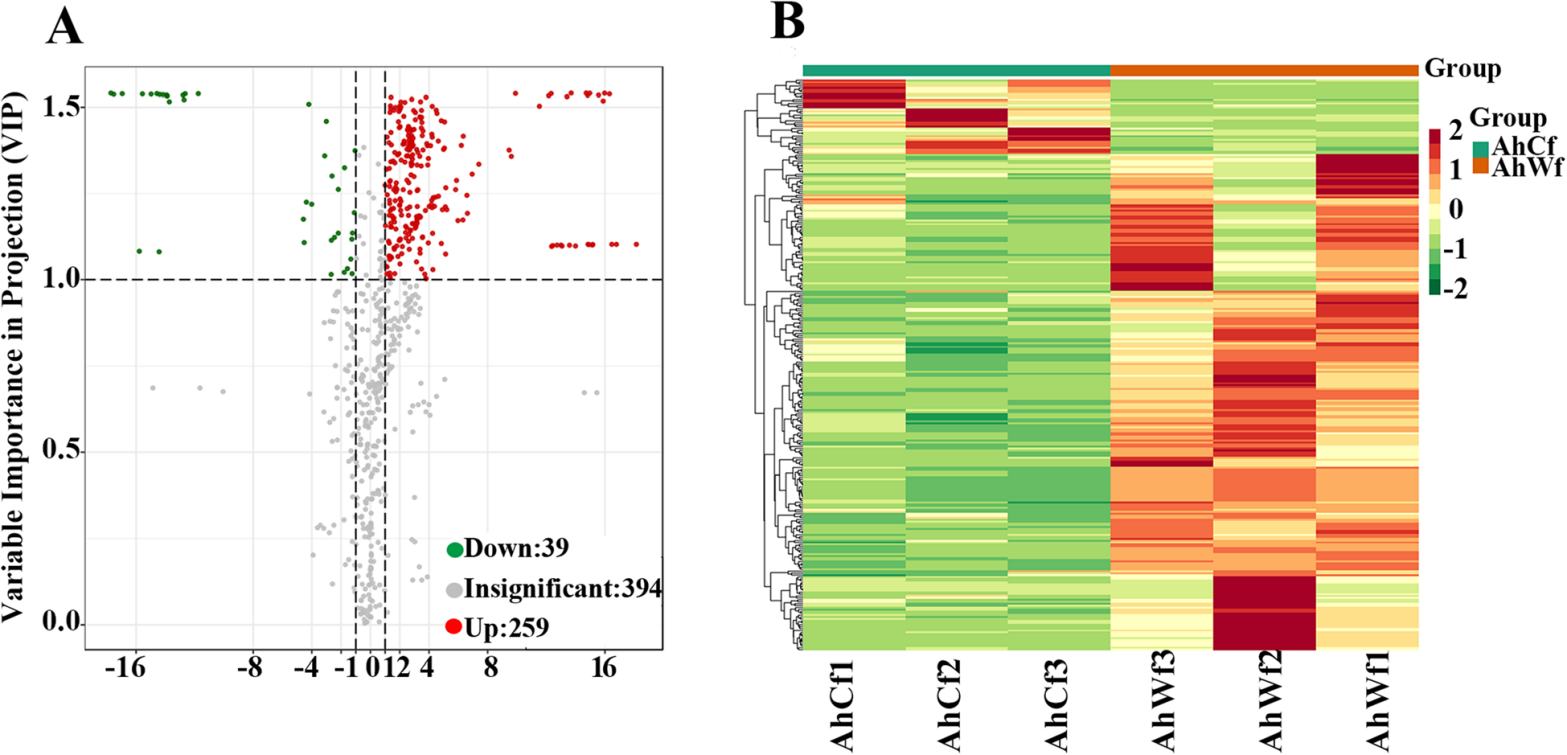
Analysis of significantly changed metabolites. **A**. Volcano plot of significantly changed metabolites (SCMs) in albino and green seedling leaves of *A. heterophyllus*. The red and green dots correspond to upregulated and downregulated metabolites, respectively. **B** Heat map of SCMs between albino and green seedlings

To compare the metabolite compositions of *A. heterophyllus* albino mutants and green seedlings, we performed metabolome analysis using a series of ultra-performance liquid chromatography (UPLC) and tandem mass spectrometry (MS/MS) experiments. Three biological replicates of leaf tissues of albino mutants and green seedlings were used for metabolic profile analysis. We identified and quantified 692 metabolites in *A. heterophyllus* seedling leaves and grouped them into 23 classes (Supplementary Table S3). We identified 298 significantly changed metabolites (SCMs) using FC ≥ 2 or FC ≤ 0.5 and variable importance in projection (VIP) ≥ 1 as thresholds. Of these SCMs, 259 were upregulated and 39 were downregulated in albino versus (vs.) green seedlings (Fig. 2A). The major SCMs included amino acids and their derivatives, flavone, organic acids and their derivatives, lipids, and phenylpropanoids.

We used the KEGG database to annotate the SCMs and analyze their metabolic pathways. KEGG enrichment analysis of the SCMs showed that the top three enriched KEGG pathways were ‘protein digestion and absorption’, ‘biosynthesis of phenylpropanoids’, and ‘flavonoid biosynthesis’ (Supplementary Fig. S3). Further analysis showed that 76 SCMs in the albino mutants were associated with metabolic pathways, and four were involved in the ‘carbon fixation in photosynthesis’ and ‘tricarboxylic acid cycle (TCA cycle)’ pathways (Supplementary Table S4). These results suggest that *A. heterophyllus* albino mutants might respond to albinism by inducing the synthesis of antioxidants and metabolites involved in carbon fixation and the TCA cycle.

### Network analysis of DEGs and SCMs related to carbon fixation and the TCA cycle in albino mutants

**Fig. 3:**
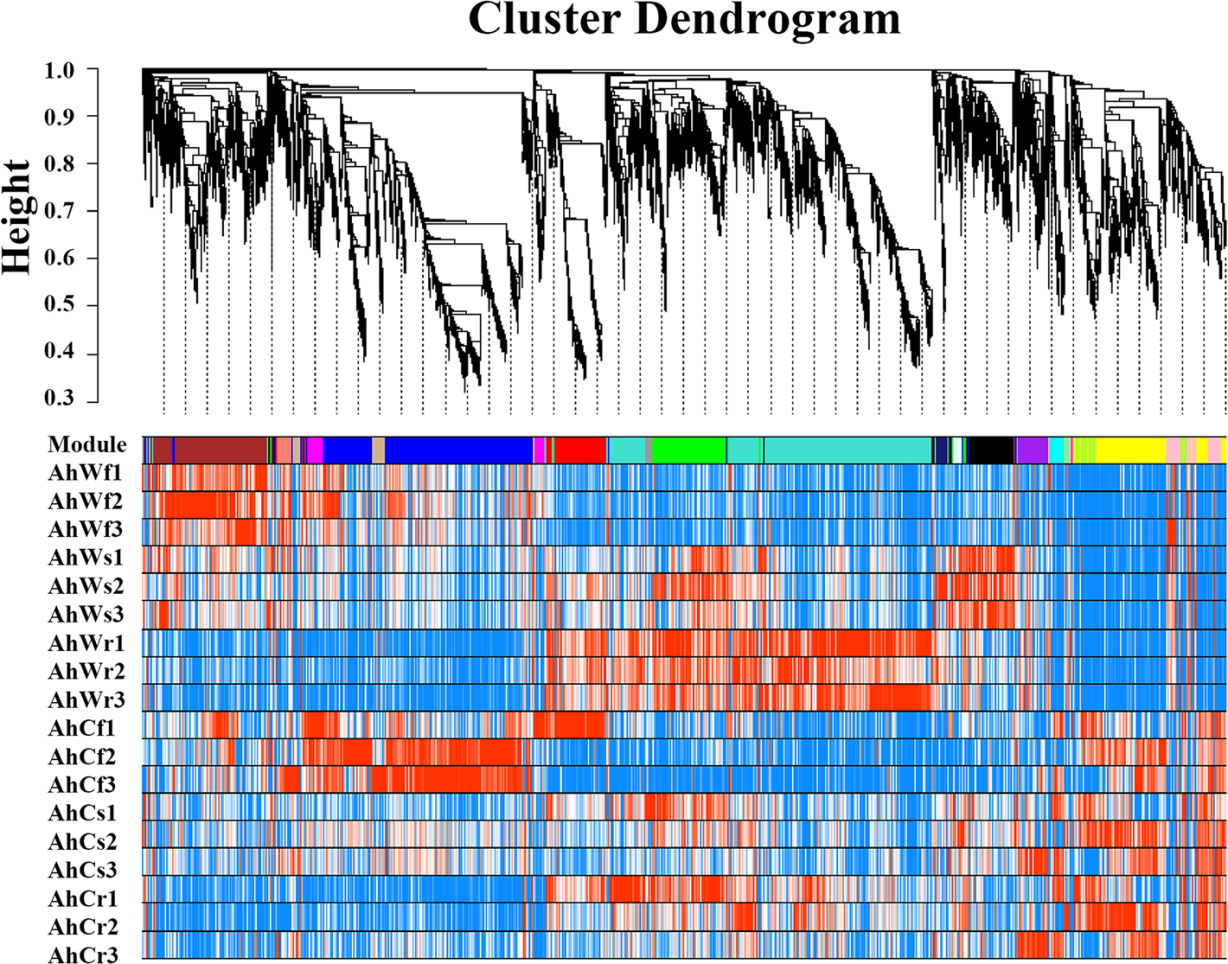
Weighted gene co-expression network analysis (WGCNA) of 8,202 DEGs. Hierarchical clustering tree (dendrogram) of genes based on coexpression network analysis of albino mutants and green seedlings. The colors were randomly assigned

**Fig. 4:**
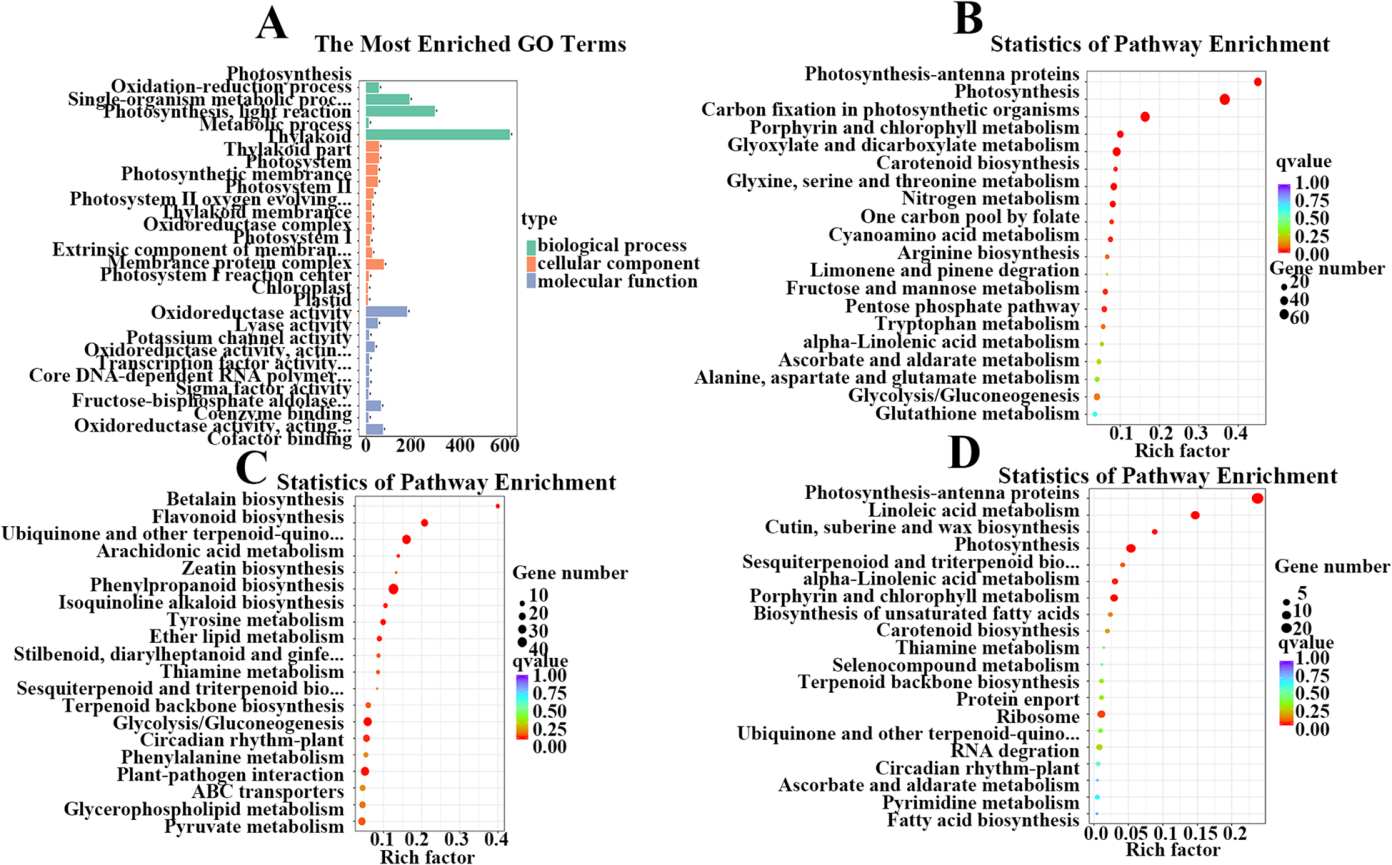
GO and KEGG enrichment analysis of genes. GO analysis of genes in the blue module (**A**); KEGG pathway enrichment analysis of genes in the blue (**B**), magenta (**C**), and turquoise (**D**) modules

To investigate the gene regulatory networks in the albino mutants, we identified co-expressed genes via WGCNA [18]. Gene regulatory network analysis revealed several major subnetworks representing interactions among genes with similar expression profiles, which are referred to as co-expression modules hereafter. In total, 8,202 DEGs were clustered into 17 modules (composed of 45–1,857 genes), which are represented by different colors (Fig. 3). Interesting pathways were also identified in the blue, magenta, and turquoise modules by GO and KEGG enrichment analysis. GO enrichment analysis of genes in the blue module showed that the ‘photosynthesis’ and ‘photosynthesis light reaction’ terms were significantly enriched (Fig. 4A). Additionally, the ‘photosynthesis’ and ‘carbon fixation in photosynthetic organisms’ pathways were significantly enriched in the blue module (Fig. 4B). The genes in the magenta module were associated with the photosynthesis process and significantly enriched in the ‘photosynthesis’ pathway (Fig. 4D). The ‘glycolysis/gluconeogenesis’ pathway was significantly enriched by KEGG enrichment analysis of genes in the turquoise module (Fig. 4C).

**Fig. 5:**
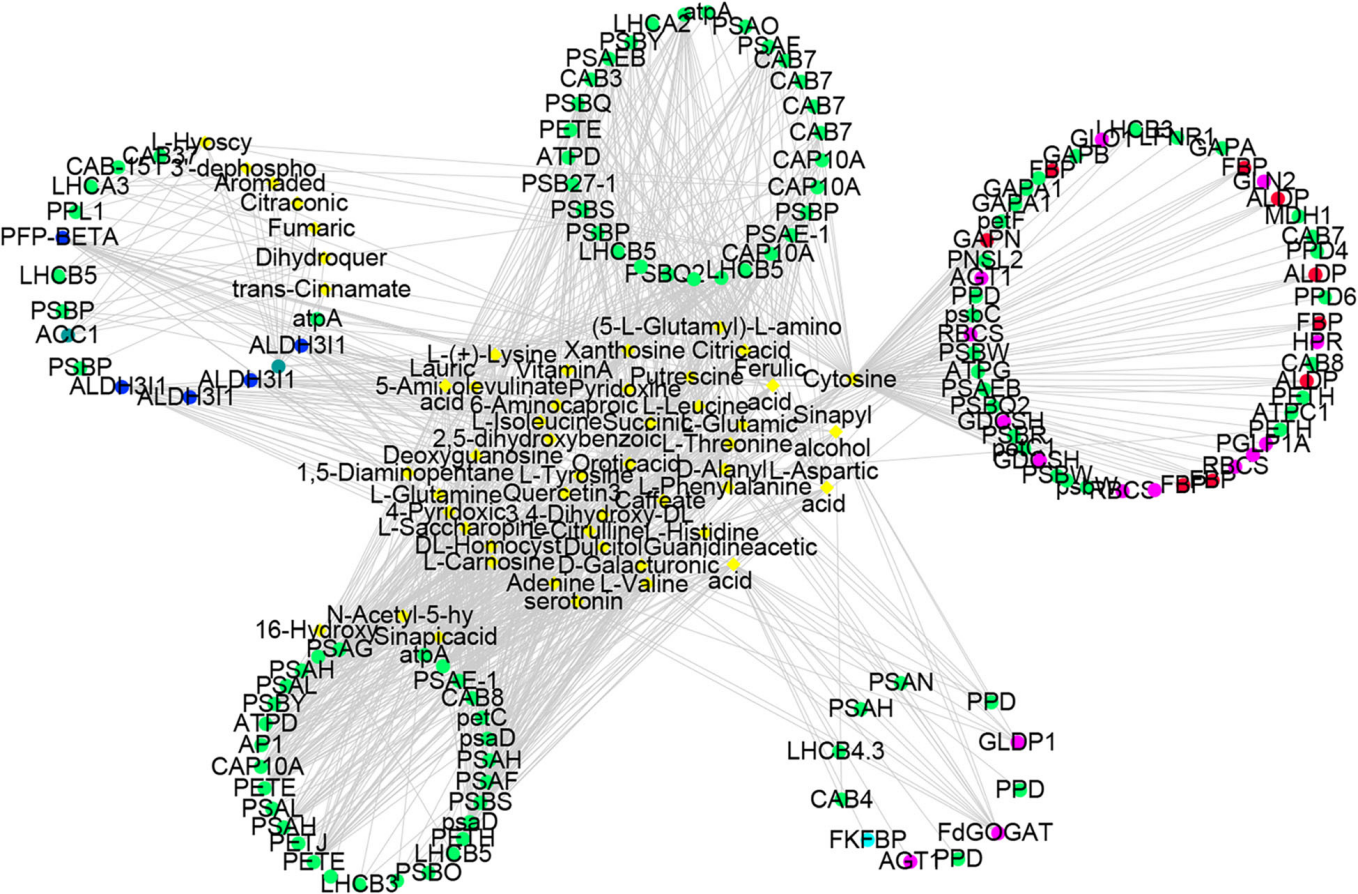
Correlation network between 333 DEGs involved in metabolic pathways and 76 SCMs. Diamonds represent significantly changed metabolites, and circles represent genes in metabolic pathways

To explore the potential correlations between genes and metabolites in various metabolic pathways, we selected 333 DEGs and 76 SCMs associated with metabolic pathways and used them to construct a correlation network by calculating Pearson correlation coefficients (Fig. 5 and Supplementary Table S4, S5). We identified 248 transcripts with extremely strong correlation coefficient values (|*R*| > 0.9) with 65 metabolites (Supplementary Table S6). Among these, the gene encoding pyruvate kinase 1 had a strong correlation with 52 metabolites, and cytosine had a strong correlation with 125 transcripts (Supplementary Table S6). Citric acid, L-aspartic acid, and succinic acid, which are involved in carbon fixation and the TCA cycle, had strong correlations with 33, 26, and 18 genes, respectively. These findings suggest that genes in *A. heterophyllus* seedlings that are up- or downregulated in response to albinism affect metabolite levels.

To further explore the effects of albinism on the expression of genes and metabolites related to carbon fixation in *A. heterophyllus*, we analyzed the interactions of DEGs and SCMs related to this process. We identified six DEGs involved in carbon fixation in the photosynthesis pathway. Among these, genes encoding malate dehydrogenase [NADP] (MDHP), malate dehydrogenase 1 (MDH1), NADP-dependent malic enzyme (MAOX), NAD-dependent malic enzyme (MAOM), and NAD-dependent malic enzyme 2 (NAD-ME2) were downregulated in the albino seedlings. By contrast, L-aspartic acid, a downstream metabolite involved in carbon fixation, was significantly upregulated in these seedlings. L-aspartic acid is a feedback inhibitor of phosphoenolpyruvate carboxylase that functions during carbon fixation. These results suggest that the downregulation of these genes and the significant upregulation of L-aspartic acid inhibit carbon fixation, thereby reducing photosynthetic efficiency and inhibiting plant growth. These results suggest that the DEGs and SCMs related to carbon fixation in the photosynthetic pathway in the albino mutants jointly inhibit carbon fixation in response to albinism.

To investigate the effects of plant albinism on the expression of genes and metabolites related to the TCA cycle, we analyzed the interactions of DEGs and SCMs related to this process. Two genes and four metabolites were found to be related to the TCA cycle in the albino mutants. The genes encoding aconitase 1 (*ACO1*) and malate dehydrogenase (*MDHP*) were downregulated in these mutants. By contrast, citric acid, succinic acid, and fumaric acid (downstream metabolites related to the TCA cycle) were significantly upregulated in the albino mutants. The downregulation of these genes might inhibit the TCA cycle, thereby reducing the energy supply, while the significantly upregulated metabolites might reduce the degree of inhibition of energy production. These results suggest that the DEGs and SCMs related to the TCA cycle in albino mutants jointly respond to albinism.

### Analysis of transcription factor genes

A total of 5,942 genes encoding TFs were identified in this work. Expression analysis of these candidate TF genes revealed that 65, 72, and 88 were differentially expressed (|log_2_FC| ≥ 1 and *q*-value < 0.05) in the roots, stems, and leaves of the albino mutants, respectively, compared to green seedlings. Of these TF genes, 6, 11, and 8 were upregulated in albino mutant roots, stems, and leaves, respectively, compared to green seedlings, whereas the others were downregulated (Supplementary Fig. S4).

**Fig. 6:**
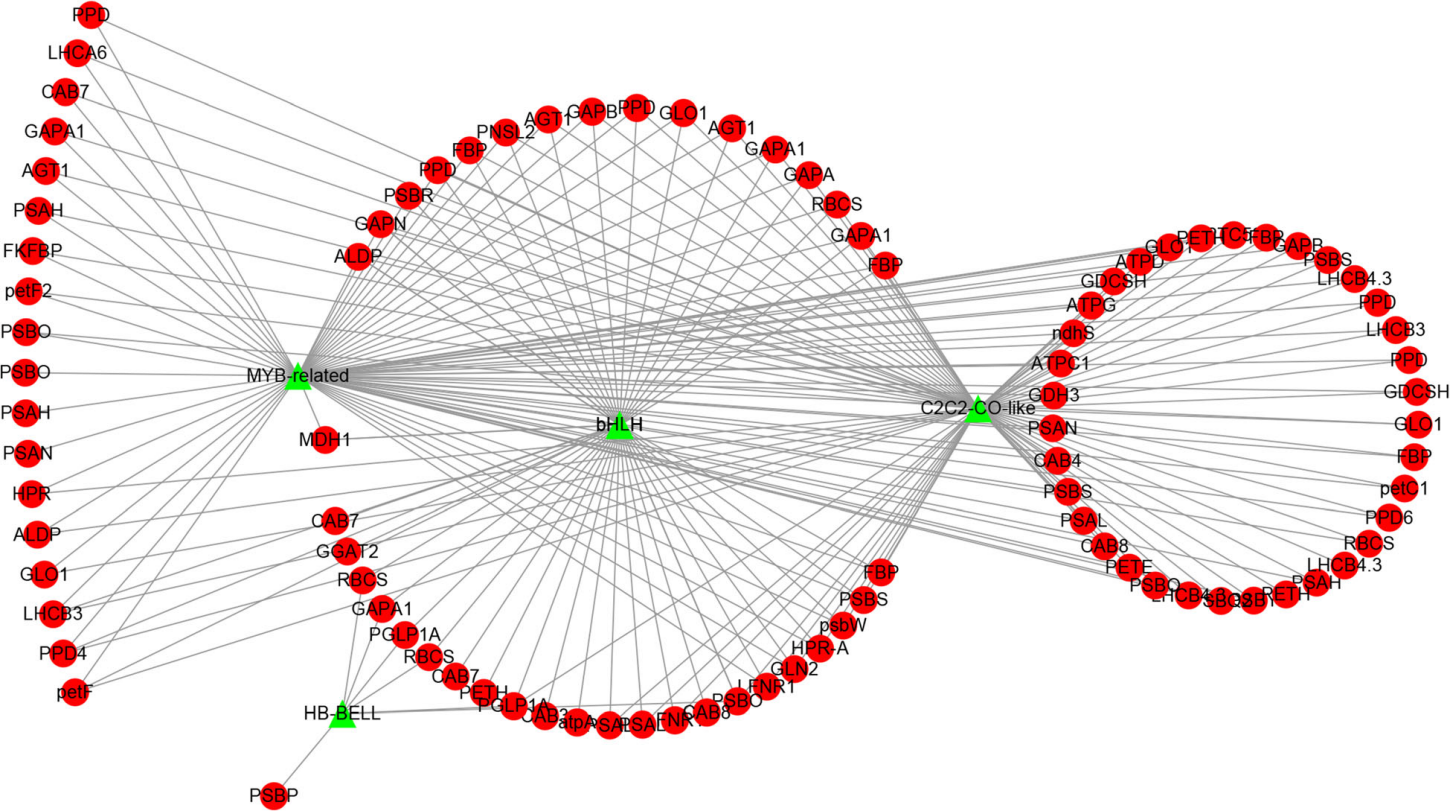
Correlation network of differentially expressed TF genes and genes involved in various metabolic pathways. Green triangles represent TFs, and red circles represent genes

We compared the expression patterns of the differentially expressed TF genes and genes involved in metabolic pathways by Pearson correlation analysis and constructed a correlation network to assess possible co-expression or co-regulation patterns in response to plant albinism (Fig. 6 and Supplementary Table S7). The most highly represented TF families in the correlation network corresponded to the MYB-related, bHLH, C2C2-CO-like, and HB-BELL TF families. Several members of these TF families (bHLH and MYB-related) were previously shown to be associated with light responses or photomorphogenesis and the circadian clock in model plant species. The bHLH TF gene *UNE10* and the MYB-related TF gene *RVE8* were identified as the hub genes in the TF-metabolic pathway gene correlation network. *UNE10* and *RVE8* were downregulated in the albino mutants, which correlated with the downregulation of the majority of metabolic pathway genes, implying that *UNE10* and *RVE8* positively regulate genes related to carbon fixation and energy metabolism.

### Validation of gene expression by qRT-PCR

**Fig. 7:**
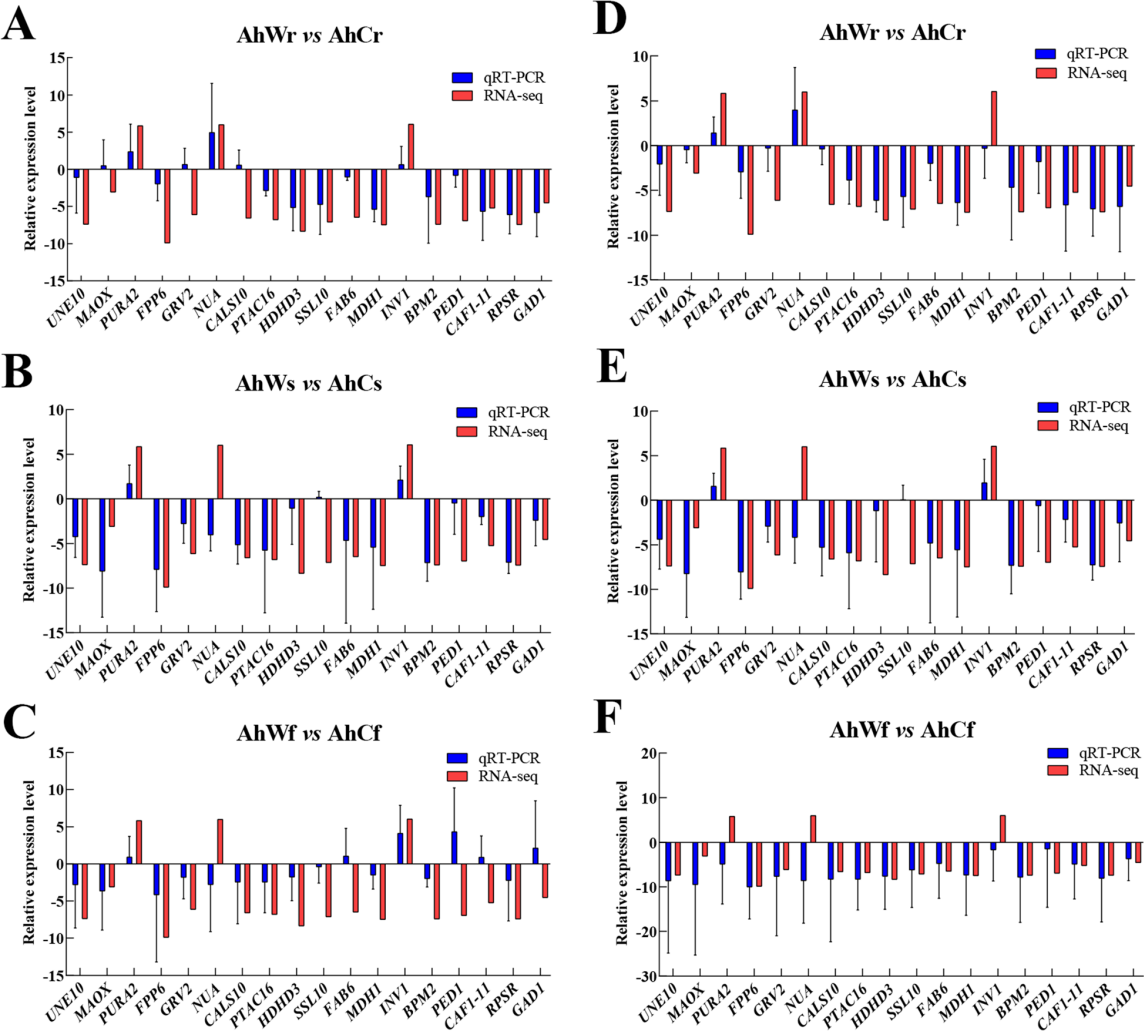
qRT-PCR of the expression levels of eighteen DEGs in the roots, stems, and leaves of albino and green seedlings. The *Actin* gene was used as the internal control in **A**, **B**, and **C**; the *Ubiquitin* gene was used as the internal control in **D**, **E**, and **F**

The expression patterns of most genes in the albino and green seedlings showed similar trends between the high-throughput sequencing data and qRT-PCR data. Although the fold change (FC) values calculated by sequencing did not exactly match the FC values detected by qRT-PCR, the expression profiles were basically consistent for all 18 genes tested (Fig. 7). In addition, the results of the correlation between qRT-PCR results and FPKM value showed that the majority of data are correlated (Supplementary Table S8). These results confirm the reliability of the gene expression values generated from the sequencing data.

## Discussion

### The effects of plant albinism on the expression of genes and metabolites

Although the draft genome of jackfruit (*A. heterophyllus*) have been published [19], the results of clean reads mapped to genome were not good as mapped to the full-length transcripts obtained by SMRT sequencing combined with Illumina sequencing (Supplementary Table S1). So we used the full-length transcripts with an average length of 2,723 bp as reference sequences for *A. heterophyllus*. The short reads generated by RNA-seq reduce the accuracy of *de novo* assembly and annotation and make bioinformatics analysis difficult [20–22]. By contrast, SMRT sequencing produces full-length transcripts, which greatly improves the accuracy of the sequencing results [23–26]. In addition, the short reads obtained from Illumina sequencing could be used to correct the long reads obtained from SMRT sequencing to compensate for the insufficient sensitivity of SMRT sequencing for detecting short sequences, as well as insertion and deletion errors, to further ensure the reliability of the sequencing results [27]. The emergence of SMRT sequencing technology from the PacBio platform has greatly facilitated the *de novo* assembly of transcriptomes in eukaryotes [14, 15].

Short reads that were previously obtained from RNA-seq of *A. heterophyllus* had an average transcript length of 836 bp [6]. In this study, we used SMRT sequencing to obtain full-length sequences from *A. heterophyllus* with an average length of 2,720 bp, which were longer than the short reads obtained by Illumina sequencing. This greatly improved the accuracy and depth of the study.

Many studies of albino mutants have produced important findings, but these studies have had some limitations. Most of these studies have focused on the causes of albinism in the mutants [28, 29], whereas few studies have explored the effects after plant albinism, such as gene expression and metabolite changes. A thorough analysis of the changes in gene expression and metabolites after plant albinism could improve the understanding of albino mutants. In this study, 8,202 DEGs were identified as responding to albinism in three different tissues, and 298 SCMs were identified in the leaves of *A. heterophyllus* albino mutants. These DEGs and SCMs provide a foundation for further research. According to WGCNA, 8,202 DEGs were clustered into 17 modules. Through GO and KEGG enrichment analysis of the genes in each module, we found that genes in the blue, turquoise, and magenta modules were significantly enriched in photosynthesis and glycolysis/gluconeogenesis pathways and other related processes. We also found that L-aspartic acid, citric acid, succinic acid, and fumaric acid levels significantly increased in the albino mutants. Further research on these DEGs and SCMs should shed light on the relationship between genes and metabolites and help identify the genes and metabolites that function in the plant response to albinism.

### Changes in *UNE10* and *RVE8* expression inhibit the light response and impair the circadian clock in plants

Development is based on the cellular capacity for differential gene expression. This, in turn, is often controlled by TFs, which function as switches in regulatory cascades [30]. bHLH TF family genes are associated with light responses or photomorphogenesis [31]. Among the many environmental factors that influence plant development, light is one of the most critical [32]. *UNE10* encodes a bHLH TF that functions as a phytochrome interacting factor. Changes in *UNE10* expression affect phytochrome A-mediated far-red light responses, thereby affecting photomorphogenesis in plants [31, 33, 34]. Therefore, changes in *UNE10* expression might play an important role in regulating the light response and photomorphogenesis processes in *A. heterophyllus* seedlings in response to albinism.

*RVE8* encodes a MYB-related TF. Changes in *RVE8* expression play an important role in regulating the circadian clock, as revealed in model plant species [35–37]. Inducing the expression of *RVE8* directly activates evening-phased genes and indirectly represses morning-phased genes. However, inhibiting *RVE8* expression leads to an extremely long circadian period, with delayed and reduced expression of evening-phased clock genes [35]. Therefore, perhaps the downregulated expression of *RVE8* impairs the circadian clock in *A. heterophyllus* albino mutants, leading to metabolic disorders and affecting normal growth.

### L-aspartic acid functions as a feedback inhibitor, and downregulated genes inhibit carbon fixation

Plants use the CO_2_ produced by self-respiration and CO_2_ in the atmosphere for carbon fixation to synthesize the carbohydrates needed for growth and development [38, 39]. In this study, L-aspartic acid was significantly upregulated and eight genes in the carbon fixation pathway were identified as differentially expressed in the albino mutant. L-aspartic acid functions as a feedback inhibitor of phosphoenolpyruvate carboxylase during carbon fixation [40]. These findings indicate that the significant upregulation of L-aspartic acid inhibits the activity of phosphoenolpyruvate carboxylase, thereby inhibiting carbon fixation. Obstructions to the synthesis of primary metabolites might cause growth to slow or even lead to death, as primary metabolites are essential for growth and reproduction [41].

Three genes encoding NAD(P)-dependent malic enzyme (*MAOX*, *MAOM*, and *NAD-ME2*) are all indispensable for carbon fixation [42, 43]. These genes encode enzymes with important roles in catalyzing the oxidative decarboxylation of L-malate to produce pyruvate and CO_2_ [44] and in releasing the CO_2_ in mesophyll cells [45]. In this study, these three genes were downregulated more than 4-fold, and *NAD-ME2* was downregulated more than 256-fold in the albino mutants compared to green *A. heterophyllus* seedlings. Perhaps the release of CO_2_ is inhibited in these mutants due to the downregulation of the candidate genes.

In summary, we propose that during carbon fixation, the efficiency of CO_2_ fixation and CO_2_ release are inhibited in the albino mutants, suggesting that insufficient materials are produced for plant growth and development. This might be the cause of the premature death of *A. heterophyllus* albino mutants. This hypothesis is consistent with the finding that photosynthesis was inhibited in an albino rice mutant, leading to death [29, 46].

### The downregulation of *ACO1* might cause dwarfing in *A. heterophyllus* albino mutants

The TCA cycle plays a crucial role in cell energy metabolism and ensures the supply of the materials and energy needed for the growth and development of organisms [47]. In this study, the DEGs involved in the TCA cycle were all downregulated, and *ACO1* was downregulated more than 512-fold in the albino mutants compared to green seedlings. *ACO1* is thought to play a role in determining plant height, as the upregulation of *ACO1* in rice is associated with internode elongation [48]. Therefore, we propose that the downregulation of *ACO1* is a key factor in the dwarf phenotype of the *A. heterophyllus* albino mutants. *ACO1* encodes aconiticase 1, the first enzyme involved in the TCA cycle, which catalyzes the conversion of citric acid to *cis*-aconitic acid. In *A. heterophyllus* albino mutants, *ACO1* expression was downregulated and citric acid was significantly upregulated, but the content of cis-aconitic acid was not significantly altered. These findings suggest that the downregulation of *ACO1* inhibits the conversion of citric acid to cis-aconitic acid, thereby disrupting the TCA cycle and inhibiting energy production in the albino mutants.

## Conclusions

In this study, third-generation sequencing technology provided 82,572 full-length transcript sequences that could be used as reference sequences to accurately examine the transcriptome of *A. heterophyllus*. In total, 8,202 DEGs were identified in *A. heterophyllus* albino mutants compared to green seedlings. Moreover, 298 SCMs were detected in the leaves using UPLC-MS/MS. The pathways ‘carbon fixation of photosynthesis’ and ‘TCA cycle’ were significantly enriched after plant albinism, as determined by analyzing the DEGs and SCMs in roots, stems, and leaves of albino mutants versus green seedlings. Comparative transcriptional and metabolic analysis revealed novel candidate genes that might play regulatory and functional roles in carbon fixation and the TCA cycle in *A. heterophyllus* seedlings in response to albinism. Our study identified candidate genes and metabolites after *A. heterophyllus* seedling albinism, laying the foundation for further analysis of the regulatory mechanisms of carbon fixation and the TCA cycle. In addition, our findings expand the understanding of albino mutants and enrich the available data for tropical fruit trees.

## Methods

### Plant materials

We accidentally discovered the albino mutants in the offspring of an *A. heterophyllus* tree. The albino mutants’ characteristics were obvious, including white leaves and stems, but the seeds showed no obvious differences from the seeds producing non-albino seedlings. Fu et al. conducted the morphological observation and determined the physiological indices of jackfruit albino mutants. The results showed that the chlorophyll content of jackfruit albino mutants was lower than that in green seedlings, while the water content, transpiration rate, and proline content were higher than those in green seedlings [8]. The seeds were collected in June 2018 and were sown in an experimental greenhouse at Hainan University (Danzhou; 109°29′25″ E, 19°30′40″ N) (Supplementary Fig. S5). Nine seedlings at the six-leaf stage showed chlorosis or complete albinism and were selected as the albino experimental materials. Green seedlings at the six-leaf stage were selected as the control.

The roots, stems, and leaves of the seedlings were used for analysis. The samples were named AhWr (root), AhWs (stem), and AhWf (leaf) for *A. heterophyllus* albino seedlings and AhCr (root), AhCs (stem), and AhCf (leaf) for green seedlings (control). The leaves and stems of the *A. heterophyllus* albino mutants were white, and the plants grew normally before the six-leaf stage (Supplementary Fig. S5). The collected samples were immediately frozen in liquid nitrogen and stored at −80 °C for RNA isolation. Each sample had three biological replicates. Eighteen samples (AhWr1, AhWr2, AhWr3; AhWs1, AhWs2, AhWs3; AhWf1, AhWf2, AhWf3; AhCr1, AhCr2, AhCr3; AhCs1, AhCs2, AhCs3; AhCf1, AhCf2, and AhCf3) were used for RNA extraction, and total RNA from the 18 samples was used for Illumina sequencing. For SMRT sequencing, total RNA from the 18 samples was mixed for subsequent experiments, according to previous studies [49–52].

### RNA isolation and Illumina sequencing

Total RNA was extracted from *A. heterophyllus* roots, stems, and leaves using the cetyltrimethyl ammonium bromide (CTAB) method [53]. The samples were treated with DNase to eliminate any genomic DNA. The quality of the 18 RNA samples was assessed using a NanoDrop 2000 (Thermo Scientific) and Agilent 2100 Bioanalyzer (Agilent Technologies). We used RNA samples with OD_260/280_ ratios of 1.8 to 2.2, OD_260/230_ ratio ≥ 2, and RNA integrity number (RIN) > 6.8 for follow-up experiments. Polyadenylated mRNA was enriched using oligo(dT) magnetic beads.

For Illumina sequencing, fragmentation buffer was added to break the mRNA into shorter pieces. We synthesized single-stranded cDNA from the mRNA using random hexamer primers and synthesized double-stranded cDNAs by adding buffer, dNTPs, and DNA polymerase I. The double-stranded cDNA was purified using AMPure XP beads and subjected to end repair, the addition of the poly-A tail, ligation of the sequencing linker, and fragment size selection. Finally, the 18 cDNA libraries were subjected to PCR enrichment and sequenced on the Illumina HiSeq 2500 platform.

### PacBio Iso-Seq library preparation

To generate the SMRTbell libraries, we combined equal amounts of total RNA from the biological replicates and generated an RNA pool for SMRT sequencing. From this pool, oligo(dT) was used to enrich for mRNA containing a poly-A tail, and the mRNA was reverse transcribed into cDNA using a SMARTer PCR cDNA Synthesis Kit. We used PCR to amplify the cDNAs. The fragments were then screened for large-scale PCR to obtain sufficient cDNA. The resulting full-length cDNA was subjected to injury repair, end-repair, ligated to SMRT dumbbell-type linkers, and used to construct a full-length transcriptome library. We removed the unligated linker sequences at both ends of the cDNA, added primers, and used DNA polymerase to form a complete SMRTbell library.

The library was sequenced using the PacBio Sequel II System and SMRT. The raw Iso-Seq data were processed with SMRTlink v6.0 software to obtain subread sequences. CCSs were obtained following correction between subreads. Full-length sequences containing a 5′ primer, a 3′ primer, and a poly-A tail were clustered using the Iterative Isomer Clustering (ICE) algorithm. Finally, the resulting consensus sequences were calibrated using the clean reads to obtain high-quality sequences for subsequent analysis.

### Sample preparation, metabolite extraction, and metabolite data analysis

In jackfruit albino seedlings, the first and most obvious part of the albino phenomenon is the leaves (Supplementary Fig. S5). This might mean that the metabolites of jackfruit leaves are the first to change, and changes a lot. Therefore, we determined the metabolites of jackfruit leaves to analyze the changes of metabolites between albino and green seedlings. Sample preparation, analysis of extracts, and metabolite identification and quantification were performed by Wuhan MetWare Biotechnology Co., Ltd. (http://www.metware.cn) following their standard procedures as previously described [54–56]. The frozen samples were crushed using a mixer mill (MM 400, Retsch) with zirconia beads for 1.5 min at 30 Hz. Approximately 100 mg of powder was weighed and extracted overnight at 4 °C with 1 ml aqueous methanol. Following centrifugation at 10,000 g for 10 min, the extracts were absorbed (CNWBOND Carbon-CCB SPE Cartridge, 250 mg, 3 ml; ANPEL, Shanghai, China, http://www.anpel.com.cn/cnw) and filtered (SCAA-104, 0.22-µm pore size; ANPEL, Shanghai, China, http://www.anpel.com.cn/) prior to liquid chromatography tandem mass spectrometry (LC-MS/MS) analysis.

Metabolite data analysis was conducted with Analyst 1.6.3 software (AB SCIEX, Ontario, Canada). The supervised multivariate method, partial least squares-discriminant analysis (PLS-DA), and orthogonal partial least squares-discriminant analysis (OPLS-DA) were used to maximize the metabolome differences between each pair of samples. The relative importance of each metabolite to the PLS-DA model was checked by calculating the variable importance in projection (VIP). Metabolites with VIP ≥ 1 and |log_2_ fold change (FC)| ≥ 1 were considered to be differential metabolites for group discrimination [57].

### Transcriptome profiling of albino and green seedlings

Clean reads were obtained by removing low-quality sequence fragments caused by instrument errors, reads with low overall quality, 3′ ends with base 10 quality score of Q < 20 (Q = −10log^error_ratio^), reads containing N blur, any adapter sequences, and any sequences with < 20 nucleotides. The clean reads were aligned to the ref sequence. The read count of each gene was obtained by mapping the clean reads to the ref sequence. The read counts were converted into fragments per kilobase of exon model per million mapped reads (FPKM) values.

DEGs were selected based on the criteria |log_2_FC| ≥ 1 and q-value < 0.05. All DEGs were mapped to individual terms in the Gene Ontology (GO) database (http://www.geneontology.org/), and the number of genes per term was calculated. GO enrichment analysis was then performed using GOseq software to identify significantly enriched terms in the DEGs. Analysis of gene regulatory pathways was conducted using the Kyoto Encyclopedia of Genes and Genomes (KEGG) Pathway database (http://www.genome.jp/kegg/pathway.html).

### Construction of correlation networks

Co-expression network analysis was performed in R studio using the weighted gene co-expression network analysis (WGCNA) package [18]. GO and KEGG enrichment analysis were performed on the genes in each module. The Pearson correlation coefficients between genes and TFs and the metabolites were calculated using R (version 4.0.1) (Supplementary Table S9). The interaction networks between genes and TFs and metabolites were visualized using Cytoscape (version 3.7.2).

### Validation by quantitative Reverse-Transcription PCR (qRT-PCR)

cDNAs were synthesized by reverse transcription of total RNA from 18 *A. heterophyllus* samples (AhWr1, AhWr2, AhWr3; AhWs1, AhWs2, AhWs3; AhWf1, AhWf2, AhWf3; AhCr1, AhCr2, AhCr3; AhCs1, AhCs2, AhCs3; AhCf1, AhCf2, and AhCf3). Primer Premier v5 software was used to design specific primers for the target genes (Supplementary Table S10). Eighteen DEGs in the roots, stems, and leaves of green and albino *A. heterophyllus* seedlings were chosen. For the latter, TB Green Premix Ex Taq II (Tli RNaseH Plus; Takara, Beijing, China) was used for qRT-PCR analysis following the manufacturer’s recommendations. PCR amplification was performed at 95 °C for 30 s for 40 cycles. The *Actin* and *Ubiquitin* genes served as internal controls for normalization (Supplementary Table S10. The expression levels of the DEGs were calculated using the 2^−△△Ct^ method against internal control gene [58]. Three technical replicates per sample were analyzed to ensure reproducibility and reliability.

## Supplementary Information

**Additional file 1: Supplementary Figure S1.** GO enrichment analysis of genes in six clusters. GO enrichment analysis of cluster 1 (A), cluster 2 (B), cluster 3 (C), cluster 4 (D), cluster 5 (E), and cluster 6 (F). **Supplementary Figure S2.** KEGG pathway analysis of genes in six clusters. KEGG pathway analysis of cluster 1 (A), cluster 2 (B), cluster 3 (C), cluster 4 (D), cluster 5 (E), and cluster 6 (F). **Supplementary Figure S3.** KEGG pathway analysis of metabolites. **Supplementary Figure S4.** Heat map of differentially expressed TF genes in *A. heterophyllus*.Roots (A), stems (B), and leaves (C). Red and blue correspond to upregulated and downregulated genes, respectively. **Supplementary Figure S5.** The growth status of *A. heterophyllus* albino mutants and green seedlings*.*

**Additional file 2: Supplementary Table S1.** Results of reads alignment to the PacBio full length transcripts and jackfruit draft genome. **Supplementary Table S9.** R code used to calculate the Pearson correlation coefficients. **Supplementary Table S10.** Oligonucleotide primers used in qRT-PCR assays in this study.

**Additional file 3: Supplementary Table S2.** The DEGs from the comparison between every two tissues.

**Additional file 4: Supplementary Table S3.** List of all metabolites identified in albino and green seedling leaves. **Supplementary Table S4.** List of significantly changed metabolites involved in different metabolic pathways. **Supplementary Table S5.** Annotation of transcripts involved in carbon fixation and energy metabolism. **Supplementary Table S6.** Pearson correlation coefficients between genes and metabolites. **Supplementary Table S7.** Pearson correlation coefficients between metabolic pathway genes and transcription factor genes. **Supplementary Table S8.** The correlation between qRT-PCR results and FPKM value. root_qRT-PCR, stem_qRT-PCR and leaf_qRT-PCR mean the qRT-PCR results of root, stem and leaf; root_RNA-seq, stem_RNA-seq and leaf_RNA-seq mean the log_2_fold change results of RNA-seq of root, stem and leaf.

## Data Availability

The raw sequence data reported in this paper have been deposited in the Genome Sequence Archive [59] in BIG Data Center (BIG Data Center Members 2019), Beijing Institute of Genomics (BIG), Chinese Academy of Sciences, under accession numbers CRA002905 and CRA003273, which are publicly accessible at https://bigd.big.ac.cn/gsa.

## Abbreviations

SMRT: Single-molecule real-time
RNA-seq: RNA-sequencing
DEGs: Differentially expressed genes
TF: Transcription factor
SCMs: Significantly changed metabolites
CTAB: Cetyltrimethyl ammonium bromide
RIN: RNA integrity number
CCS: Circular consensus sequence
ICE: Iterative Isomer Clustering
LC-MS/MS: Liquid chromatography tandem mass spectrometry
PLS-DA: Partial least squares-discriminant analysis
OPLS-DA: Orthogonal partial least squares-discriminant analysis
VIP: Variable importance in projection
FC: Fold change
FPKM: Fragments per kilobase of exon model per million mapped reads
GO: Gene Ontology
KEGG: Kyoto Encyclopedia of Genes and Genomes
WGCNA: Weighted gene co-expression network analysis
FLNC: Full-length non-chimeric
ref: Reference
UPLC: Ultra-performance liquid chromatography
MS/MS: Tandem mass spectrometry
SCMs: Significantly changed metabolites
TCA cycle: Tricarboxylic acid cycle
NAD-ME2: NAD-dependent malic enzyme 2
qRT-PCR: Quantitative Reverse-Transcription Polymerase Chain Reaction
vs.: Versus

## References

[1] Zhang Q, Tang D, Liu M, Ruan J (2018). Integrated analyses of the transcriptome and metabolome of the leaves of albino tea cultivars reveal coordinated regulation of the carbon and nitrogen metabolism. Sci Hortic.

[2] Li Y, Li W, Hu D, Shen P, Zhang G, Zhu Y (2020). Comparative analysis of the metabolome and transcriptome between green and albino zones of variegated leaves from *Hydrangea macrophylla* ‘Maculata’ infected by hydrangea ringspot virus. Plant Physiol Biochem.

[3] Satou M, Enoki H, Oikawa A (2014). Integrated analysis of transcriptome and metabolome of *Arabidopsis albino or pale green* mutants with disrupted nuclear-encoded chloroplast proteins. Plant Mol Biol.

[4] Tang J, Zhang W, Wen K (2017). OsPPR6, a pentatricopeptide repeat protein involved in editing and splicing chloroplast RNA, is required for chloroplast biogenesis in rice. Plant Mol Biol.

[5] Yu QB, Jiang Y, Chong K, Yang ZN (2009). AtECB2, a pentatricopeptide repeat protein, is required for chloroplast transcript accd rna editing and early chloroplast biogenesis in *Arabidopsis thaliana*. Plant J.

[6] Hu L, Wu G, Hao C, Yu H, Tan L (2016). Transcriptome and selected metabolite analyses reveal points of sugar metabolism in jackfruit (*Artocarpus heterophyllus* Lam.). Plant Sci.

[7] Baliga MS, Shivashankara AR, Haniadka R, Dsouza J, Bhat HP (2011). Phytochemistry, nutritional and pharmacological properties of *Artocarpus heterophyllus* Lam (jackfruit): A review. Food Res Int.

[8] Fu Y, Yu X, Cai Z, Wu F, Luo J (2018). Characters of Albino Mutant of *Artocarpus heterophyllus* Lam. Chinese Journal of Tropical Crops.

[9] Xie L, Dong J, Yu X, Cai Z, Luo J, Jiang S (2020). Transcriptome Analysis of Stem Secondary Growth in Chlorophyll Deficient Mutant of *Artocarpus heterophyllus*. Mol Plant Breed China..

[10] Cho K, Cho KS, Sohn HB (2016). Network analysis of the metabolome and transcriptome reveals novel regulation of potato pigmentation. J Exp Bot.

[11] Meng J, Wang B, He G (2019). Metabolomics Integrated with Transcriptomics Reveals Redirection of the Phenylpropanoids Metabolic Flux in *Ginkgo biloba*. J Agric Food Chem.

[12] Li Y, Chen Y, Zhou L (2020). MicroTom Metabolic Network: Rewiring Tomato Metabolic Regulatory Network throughout the Growth Cycle. Mol Plant.

[13] Yang L, Jin Y, Huang W, Sun Q, Liu F, Huang X (2018). Full-length transcriptome sequences of ephemeral plant *Arabidopsis pumila* provides insight into gene expression dynamics during continuous salt stress. BMC Genomics.

[14] Au KF, Sebastiano V, Afshar PT (2013). Characterization of the human ESC transcriptome by hybrid sequencing. Proc Natl Acad Sci USA.

[15] Sharon D, Tilgner H, Grubert F, Snyder M (2013). A single-molecule long-read survey of the human transcriptome. Nat Biotechnol.

[16] Guo J, Wu Y, Wang G, Wang T, Cao F (2020). Integrated analysis of the transcriptome and metabolome in young and mature leaves of *Ginkgo biloba* L. Ind Crops Prod.

[17] Saito K, Matsuda F (2010). Metabolomics for functional genomics, systems biology, and biotechnology. Annu Rev Plant Biol.

[18] Zhang B, Horvath S (2005). A general framework for weighted gene co-expression network analysis. Stat Appl Genet Mol Biol..

[19] Sahu SK, Liu M, Yssel A (2020). Draft genomes of two artocarpus plants, jackfruit (*A. heterophyllus*) and breadfruit (*A. altilis*). Genes (Basel).

[20] Hackl T, Hedrich R, Schultz J, Förster F (2014). proovread: large-scale high-accuracy PacBio correction through iterative short read consensus. Bioinformatics.

[21] Nagarajan N, Pop M (2013). Sequence assembly demystified. Nat Rev Genet.

[22] Pop M, Salzberg SL (2008). Bioinformatics challenges of new sequencing technology. Trends Genet.

[23] Rhoads A, Au KF (2015). PacBio sequencing and its applications. Genomics Proteomics Bioinforma.

[24] Uemura S, Aitken CE, Korlach J, Flusberg BA, Turner SW, Puglisi JD (2010). Real-time tRNA transit on single translating ribosomes at codon resolution. Nature.

[25] Eid J, Fehr A, Gray J (2009). Real-time DNA sequencing from single polymerase molecules. Science.

[26] Levene MJ, Korlach J, Turner SW, Foquet M, Craighead HG, Webb WW (2003). Zero-mode waveguides for single-molecule analysis at high concentrations. Science.

[27] Tombacz D, Csabai Z, Olâh P (2015). Characterization of novel transcripts in pseudorabies virus. Viruses.

[28] Sundberg E, Slagter JG, Fridborg I, Cleary SP, Robinson C, Coupland G (1997). ALBINO3, an arabidopsis nuclear gene essential for chloroplast differentiation, encodes a chloroplast protein that shows homology to proteins present in bacterial membranes and yeast mitochondria. Plant Cell.

[29] Zhu M, Hu Z, Zhou S, Li Y, Chen G (2012). Research progress of plant leaf albino. Chinese Bulletin of Life Sciences.

[30] Scott MP (2000). Development: The natural history of genes. Cell.

[31] Qiu Y, Li M, Pasoreck EK (2015). Hemera couples the proteolysis and transcriptional activity of phytochrome interacting actors in *Arabidopsis* photomorphogenesis. Plant Cell.

[32] Franklin KA, Quail PH (2010). Phytochrome functions in *Arabidopsis* development. J Exp Bot.

[33] Xu Z, Peters RJ, Weirather J (2015). Full-length transcriptome sequences and splice variants obtained by a combination of sequencing platforms applied to different root tissues of *Salvia miltiorrhiza* and tanshinone biosynthesis. Plant J.

[34] Hoecker U (2017). The activities of the E3 ubiquitin ligase COP1/SPA, a key repressor in light signaling. Curr Opin Plant Biol.

[35] Hsu PY, Devisetty UK, Harmer SL (2013). Accurate timekeeping is controlled by a cycling activator in *Arabidopsis*. Elife.

[36] Farinas B, Mas P (2011). Functional implication of the MYB transcription factor RVE8/LCL5 in the circadian control of histone acetylation. Plant J.

[37] Rawat R, Takahashi N, Hsu PY, et al. REVEILLE8 and PSEUDO-REPONSE REGULATOR5 form a negative feedback loop within the arabidopsis circadian clock. PLoS Genet. 2011;7. 10.1371/journal.pgen.1001350. 10.1371/journal.pgen.1001350 PMC3069099 21483796

[38] Nilsen ET. Stem photosynthesis: extent, patterns and role in plant carbon economy. Plant Stems. Academic Press; 1995. p.223–40.

[39] Ashraf M, Harris PJC (2013). Photosynthesis under stressful environments: An overview. Photosynthetica.

[40] Paulus JK, Schlieper D, Groth G (2013). Greater efficiency of photosynthetic carbon fixation due to single amino-acid substitution. Nat Commun.

[41] Drew SW, Demain AL (1977). Effect of primary metabolites on secondary metabolism. Annu Rev Microbiol.

[42] Tronconi MA, Maurino VG, Andreo CS, Drincovich MF (2010). Three different and tissue-specific NAD-malic enzymes generated by alternative subunit association in *Arabidopsis thaliana*. J Biol Chem.

[43] Van den Bergh E, Külahoglu C, Bräutigam A (2014). Gene and genome duplications and the origin of C4 photosynthesis: Birth of a trait in the Cleomaceae. Curr Plant Biol.

[44] Tronconi MA, Gerrard Wheeler MC, Drincovich MF, Andreo CS (2012). Differential fumarate binding to *Arabidopsis* NAD+-malic enzymes 1 and – 2 produces an opposite activity modulation. Biochimie.

[45] Furbank RT, Agostino A, Hatch MD (1990). C4 acid decarboxylation and photosynthesis in bundle sheath cells of NAD-malic enzyme-type C4 plants: Mechanism and the role of malate and orthophosphate. Arch Biochem Biophys.

[46] Zhu H, Liu Y, Pan Q, et al. Identification and gene mapping of a novel rice albino lethal abl25 mutant. J Shanghai Normal Univ (Nat Sci). 2014;(43):238–44.

[47] Fernie AR, Carrari F, Sweetlove LJ (2004). Respiratory metabolism: Glycolysis, the TCA cycle and mitochondrial electron transport. Curr Opin Plant Biol.

[48] Iwamoto M, Baba-Kasai A, Kiyota S, Hara N, Takano M (2010). *ACO1*, a gene for aminocyclopropane-1-carboxylate oxidase: Effects on internode elongation at the heading stage in rice. Plant Cell Environ.

[49] Xu D, Yang H, Zhuo Z, Lu B, Hu J, Yang F (2021). Characterization and analysis of the transcriptome in *Opisina arenosella* from different developmental stages using single-molecule real-time transcript sequencing and RNA-sEq. Int J Biol Macromol.

[50] Yao S, Liang F, Gill RA (2020). A global survey of the transcriptome of allopolyploid *Brassica napus* based on single-molecule long-read isoform sequencing and Illumina-based RNA sequencing data. Plant J.

[51] Chao Q, Gao ZF, Zhang D (2019). The developmental dynamics of the *Populus* stem transcriptome. Plant Biotechnol J.

[52] Wang B, Tseng E, Regulski M, et al. Unveiling the complexity of the maize transcriptome by single-molecule long-read sequencing. Nat Commun 2016;7. 10.1038/ncomms11708. 10.1038/ncomms11708 PMC4931018 27339440

[53] Chang S, Puryear J, Cairney J (1993). A Simple and Efficient Method for Isolating RNA from Pine Trees. Plant Mol Biol Report.

[54] Zhang Q, Wang L, Liu Z (2020). Transcriptome and metabolome profiling unveil the mechanisms of *Ziziphus jujuba* Mill. peel coloration. Food Chem.

[55] Zhang H, Chen M, Wen H (2020). Transcriptomic and metabolomic analyses provide insight into the volatile compounds of citrus leaves and flowers. BMC Plant Biol.

[56] Zhang S, Ying H, Pingcuo G (2019). Identification of Potential Metabolites Mediating Bird’s Selective Feeding on *Prunus mira* Flowers. Biomed Res Int.

[57] Yuan H, Zeng X, Shi J (2018). Time-course comparative metabolite profiling under osmotic stress in tolerant and sensitive tibetan hulless barley. Biomed Res Int.

[58] Schmittgen TD, Livak KJ (2008). Analyzing real-time PCR data by the comparative CT method. Nat Protoc.

[59] Wang Y, Song F, Zhu J (2017). GSA: Genome Sequence Archive*. Genomics Proteomics Bioinforma.

