# Supplementary material for: Third-generation sequencing and metabolome analysis reveal candidate genes and metabolites with altered levels in albino jackfruit seedlings
Source: BMC Genomics. 2021 Jul 16;22:543. doi: 10.1186/s12864-021-07873-y (PMC8283932; doi:10.1186/s12864-021-07873-y)
Supplement: Supplementary file 1 — Additional file 1: Supplementary Figure S1. GO enrichment analysis of genes in six clusters. GO enrichment analysis of cluster 1 (A), cluster 2 (B), cluster 3 (C), cluster 4 (D), cluster 5 (E), and cluster 6 (F). Supplementary Figure S2. KEGG pathway analysis of genes in six clusters. KEGG pathway analysis of cluster 1 (A), cluster 2 (B), cluster 3 (C), cluster 4 (D), cluster 5 (E), and cluster 6 (F). Supplementary Figure S3. KEGG pathway analysis of metabolites. Supplementary Figure S4. Heat map of differentially expressed TF genes in A. heterophyllus.Roots (A), stems (B), and leaves (C). Red and blue correspond to upregulated and downregulated genes, respectively. Supplementary Figure S5. The growth status of A. heterophyllus albino mutants and green seedlings. [file 12864_2021_7873_MOESM1_ESM.pdf]

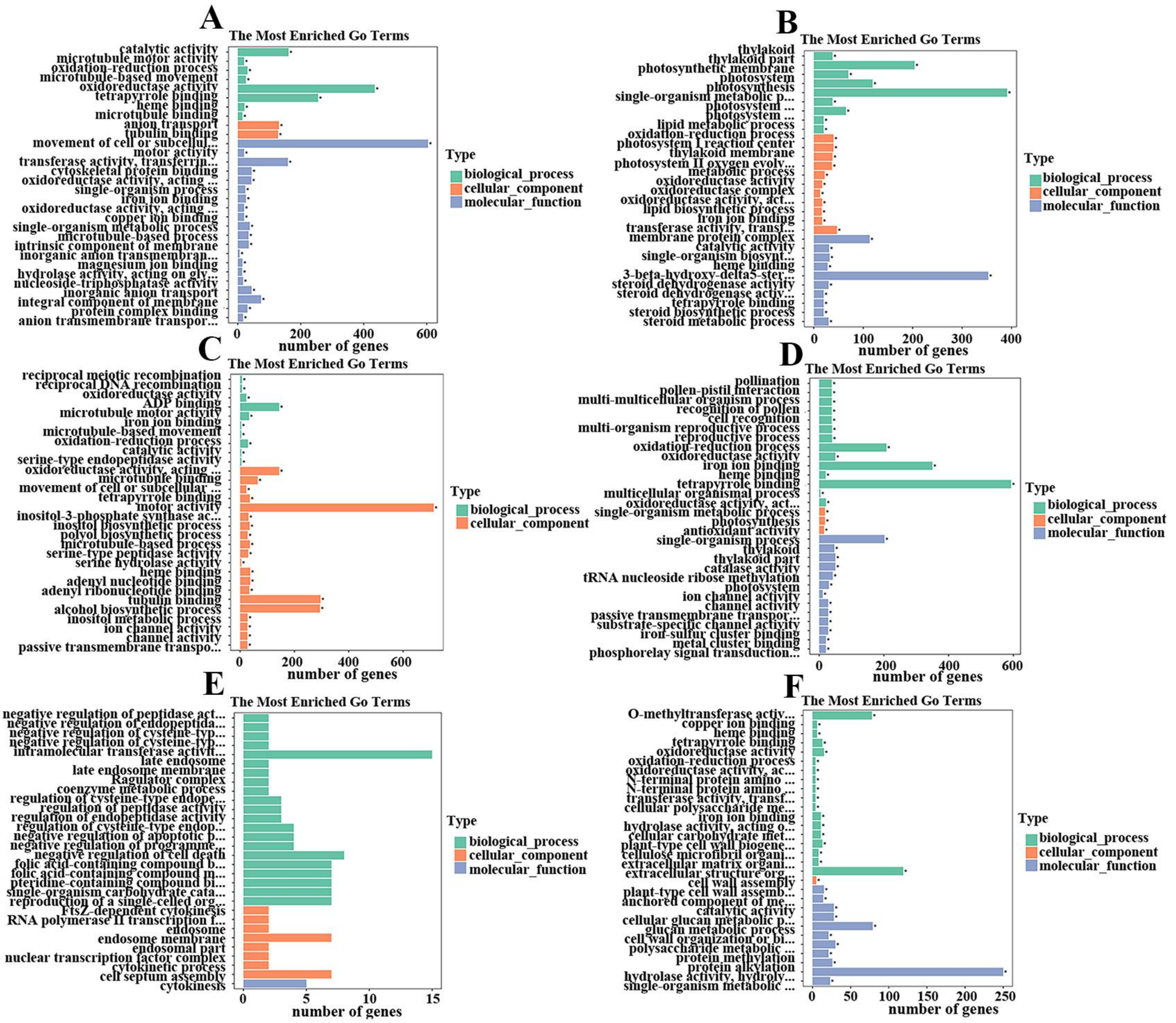

Supplementary Fig. S1 GO enrichment analysis of genes in six clusters. GO enrichment analysis of cluster 1 (A), cluster 2 (B), cluster 3 (C), cluster 4 (D), GO cluster 5 (E), and cluster 6 (F).

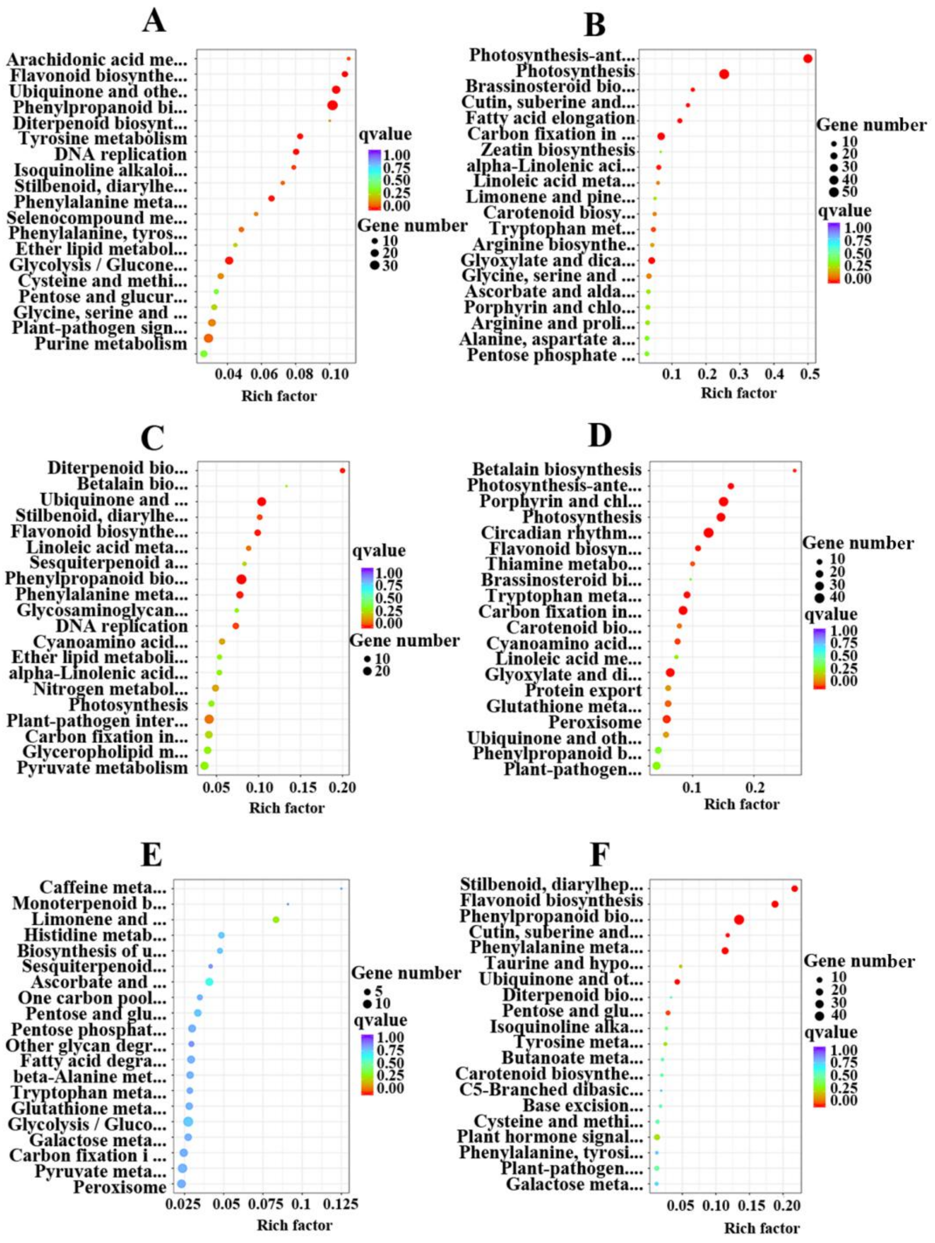

Supplementary Fig. S2 KEGG pathway analysis of genes in six clusters. KEGG pathway analysis of cluster 1 (A), **cluster 2** (B), **cluster 3** (C), **cluster 4** (D), **cluster 5** (E), and **cluster 6** (F).

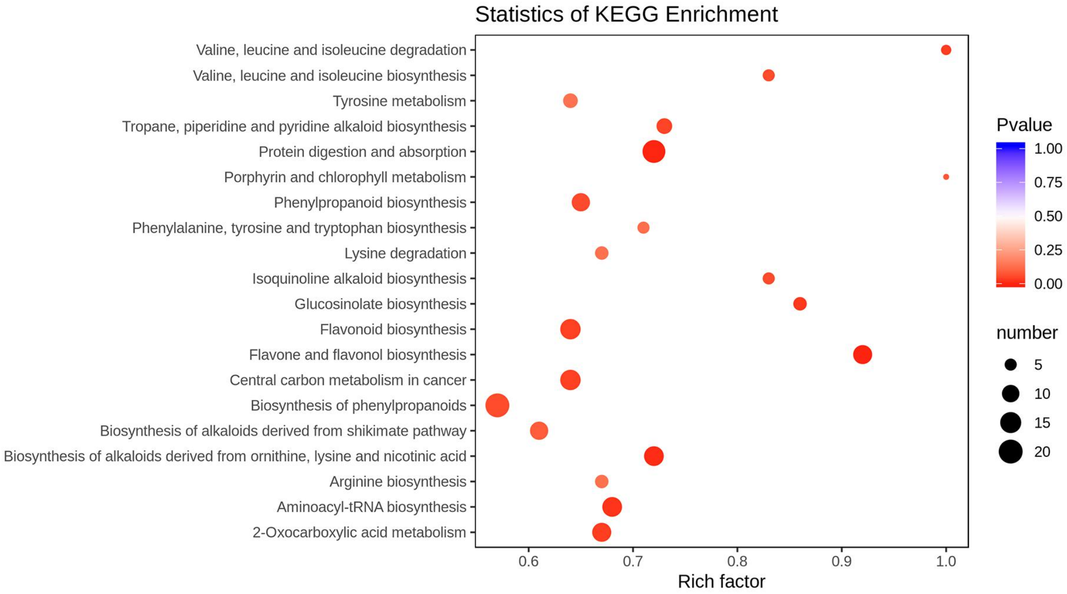

Supplementary Fig. S3 KEGG pathway analysis of metabolites.

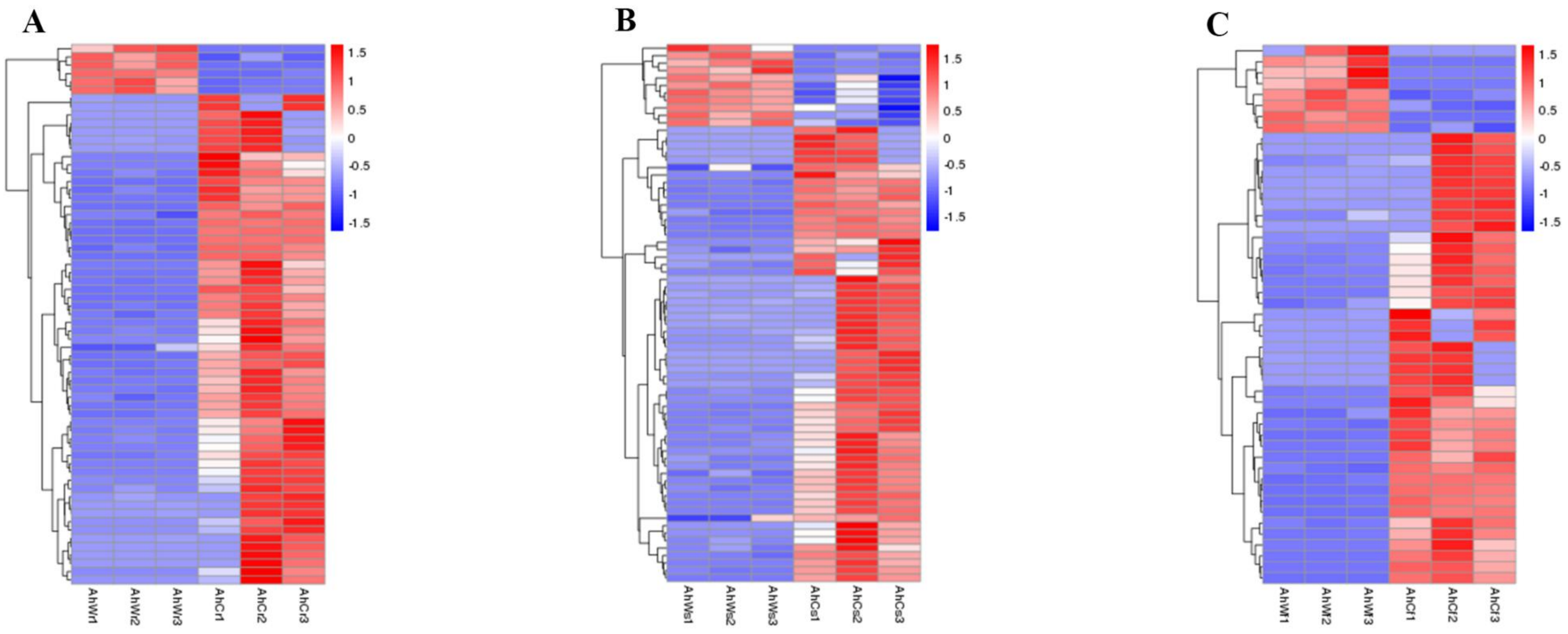

Supplementary Fig. S4. Heat map of differentially expressed TF genes in *A. heterophyllus*. Roots (A), stems (B), and leaves (C). Red and blue correspond to upregulated and downregulated genes, respectively.

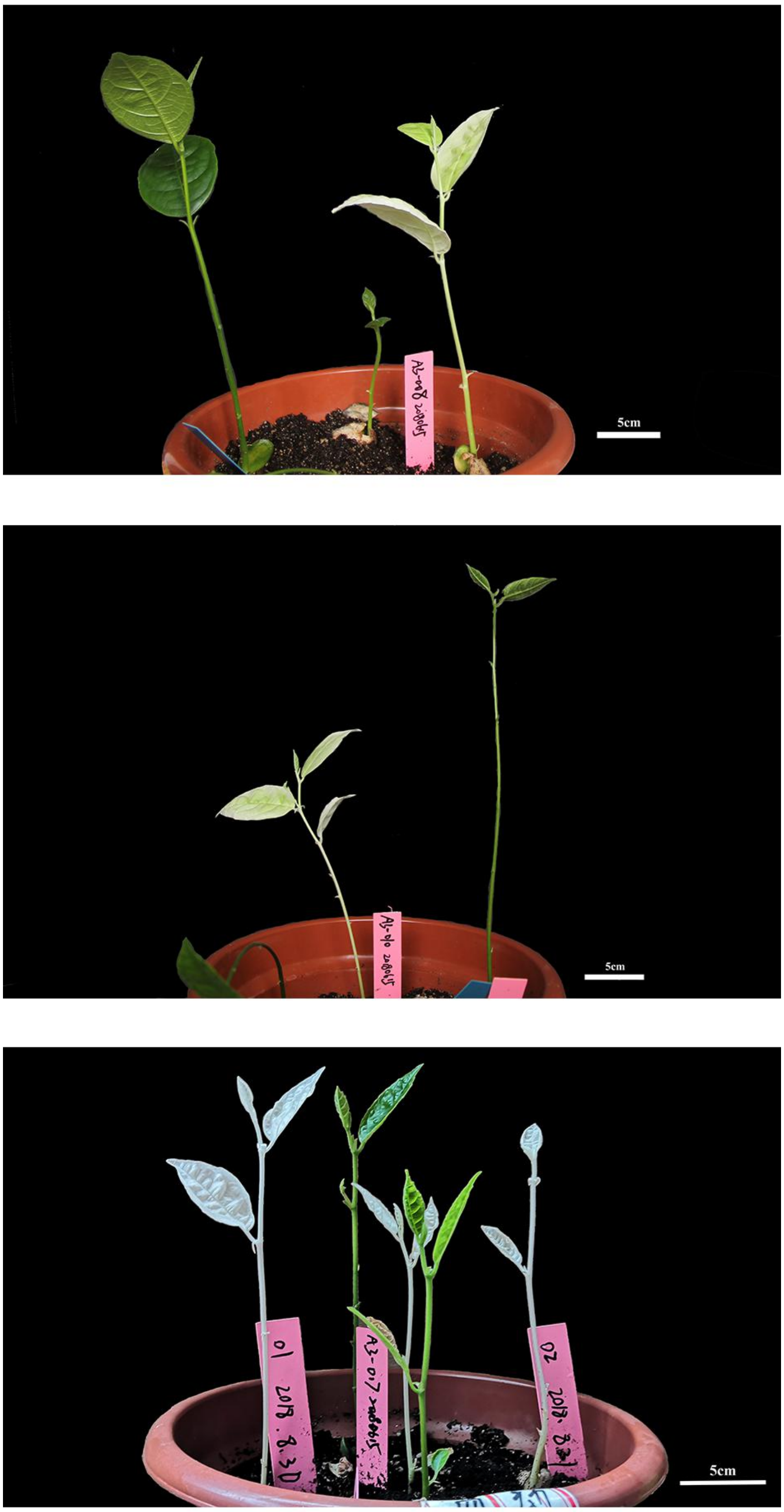

Supplementary Fig. S5. The growth status of *A. heterophyllus* albino mutants and green seedlings.
