# Supplementary material for: Third-generation sequencing and metabolome analysis reveal candidate genes and metabolites with altered levels in albino jackfruit seedlings
Source: BMC Genomics. 2021 Jul 16;22:543. doi: 10.1186/s12864-021-07873-y (PMC8283932; doi:10.1186/s12864-021-07873-y)
Supplement: Supplementary file 2 — Additional file 2: Supplementary Table S1. Results of reads alignment to the PacBio full length transcripts and jackfruit draft genome. Supplementary Table S9. R code used to calculate the Pearson correlation coefficients. Supplementary Table S10. Oligonucleotide primers used in qRT-PCR assays in this study. [file 12864_2021_7873_MOESM2_ESM.pdf]

Supplementary Table S1: Results of reads alignment to the PacBio full length transcripts and jackfruit draft genome

Sample name	Total reads	Mapped to Pacbio	Mapped to genome
AhCr1	49,624,842	38,483,936(77.55%)	33,616,341(67.74%)
AhCr2	66,242,972	54,624,870(82.46%)	46,172,639(69.70%)
AhCr3	50,938,062	40,149,002(78.82%)	34,980,762(68.67%)
AhCs1	52,473,240	43,011,438(81.97%)	38,556,092(73.48%)
AhCs2	44,375,060	36,263,786(81.72%)	31,629,199(71.28%)
AhCs3	86,213,728	72,360,092(83.93%)	62,132,131(72.07%)
AhCf1	73,301,264	59,777,600(81.55%)	53,657,523(73.20%)
AhCf2	45,827,910	39,026,306(85.16%)	32,838,901(71.66%)
AhCf3	54,180,396	45,344,338(83.69%)	38,438,992(70.95%)
AhWr1	72,461,466	59,483,124(82.09%)	52,160,891(71.98%)
AhWr2	39,423,718	31,893,036(80.90%)	28,331,577(71.86%)
AhWr3	50,012,216	41,734,824(83.45%)	36,294,711(72.57%)
AhWs1	60,132,136	50,287,642(83.63%)	43,842,553(72.91%)
AhWs2	41,567,474	34,567,956(83.16%)	30,160,571(72.56%)
AhWs3	50,063,434	41,695,770(83.29%)	37,020,198(73.95%)
AhWf1	46,200,604	38,702,746(83.77%)	33,455,412(72.41%)
AhWf2	52,369,424	43,612,690(83.28%)	37,991,591(72.55%)
AhWf3	62,345,450	52,322,688(83.92%)	45,021,552(72.21%)

Supplementary Table S9: R code used to calculate the Pearson correlation coefficients

```
setwd("      ")
a=read.table("      ",sep="\t",head=T)
a
Gene<-c("      ")
data.frame(a,row.names=1)
t1<-t(data.frame(a,row.names=1))
t1
t2<-as.data.frame(t1,row.names=F)
t2
t3<-as.data.frame(cbind(Gene,t2))
t3
library(Formula)
library(Hmisc)
head(t2)
mat=matrix(ncol=4,nrow=sum(1:(ncol(t2)-1)))
m=1
for(i in 2:(ncol(t2)-1)){
    for(j in (i+1):ncol(t2)){
        mat[m,2]=names(t2)[j]
            mat[m,1]=names(t2)[i]
            mat[m,3]=cor(t2[,i],t2[,j],method="pearson")
            w=rcorr(t2[,i],t2[,j],type="pearson")
            mat[m,4]=w$P[1,2]
        m=m+1
  }
}
colnames(mat)<-c("gene1","gene2","correlation_coefficient","pvalue")
write.table(mat,sep="\t","organ-p-value.xls",col.names=TRUE,row.names = FALSE)
```

Supplementary Table S10: Oligonucleotide primers used in qRT-PCR assays in this study

Gene	Primer pairs
Actin gene	F:TTCAGCCACTCGTTTGCGATAAT<br>R:GTGACGGGGACGACCTACAATGC
Ubiquitin gene	F:CTGATGGGCCGAGAATAGGC<br>R:AAGGCAGTCATAGAGACCCA
UNE10	F:GCTTGCCAGTCACAGCCTAT<br>R:AGTTTCGTGACGCCTCCAAG
MAOX	F:GCTAATGTTGCCGCTAAGGC<br>R:CCGCCATGACGATAGCAAGA
PURA2	F:GTCAGTGAAGCTGTTCGGGA<br>R:CCCAGAAACCCAATTGCAGC
FPP6	F:CGAAGAAGCCGTCTCAGGTT<br>R:CGAAGAAGCCGTCTCAGGTT
GRV2	F:TTCGTCAGGTCTTGCAGCAT<br>R:GAGATCCATGGGCCTTCTCG
NUA	F:AGGAACTGCATTAGCGGCTT<br>R:TGTTCCGCCCTTTCATCCAA
CALS10	F:GTGAACCCTCGATTCACGCT<br>R:AGACGCTAAGATGGTCGCAG
PTAC16	F:AAAAGCACGGGCTGCAAGTA<br>R:CTTGTCTGCCTTTCTTCGGC
HDHD3	F:AAGGGCGATCAGGACTAAGC<br>R:GACGAGCAAAGCCTTGTGAC
SSL10	F:CCGAGTCAGCCCTCTAACAC<br>R:CCGTCAGAGTCGAAAGCGAT
FAB6	F:TGAAAATGCAAACGGCCTGG

	R:CAGGGCACGAACTGTGTACT
MDH1	F:ACTCAACTACGCAGGTTCCG<br>R:TGCAAGTTCGTAGTCGCCAT
INV1	F:TGGGCCCATTCTGTGTCAAA<br>R:TTTACGCCCACATCCGGAAA
BPM2	F:CCTGTATTCCGGGCACAACT<br>R:AAACGCACAACGCATAGCAG
PED1	F:TGTCGCAGCATATCGTACCC<br>R:AAAATCCAGCCCTGATCGCA
CAF1-11	F:GCGAAGATCCGAGTCCGAAT<br>R:TCGAAGTCGCGGAAGTTGAA
RPSR	F:AACTCGGGCAACCATCAACT<br>R:AAAGGGCTCTGAGCACATGG
GAD1	F:TTGTGTCGCTGCCATTTTGG<br>R:GATCCGCTCCGAGGTAGTTG
